# Cellulose-Based Pickering Emulsion-Templated Edible Oleofoam: A Novel Approach to Healthier Solid-Fat Replacers

**DOI:** 10.3390/gels11060403

**Published:** 2025-05-28

**Authors:** Sang Min Lee, Su Jung Hong, Gye Hwa Shin, Jun Tae Kim

**Affiliations:** 1 Department of Food Science and Technology, Keimyung University, Daegu 42601, Republic of Korea; thkdals1031@naver.com; 2 Department of Food and Nutrition, Kyung Hee University, Seoul 02447, Republic of Korea; sujunghong@vt.edu; 3 Department of Sustainable Biomaterials, Virginia Tech, Blacksburg, VA 24061, USA; 4 Department of Food and Nutrition, Kunsan National University, Gunsan 54150, Republic of Korea; 5 BioNanocomposite Research Center, Kyung Hee University, Seoul 02447, Republic of Korea

**Keywords:** Pickering emulsions, edible oleofoams, solid-fat replacers, cellulose particles, oleogelation

## Abstract

As health concerns and regulatory pressures over saturated and trans fats grow, there is a growing need for healthier alternatives to traditional solid fats, such as butter and hydrogenated oils, that are still widely used in the food system. In this study, cellulose particle-based Pickering emulsions (CP-PEs) were prepared from microcrystalline cellulose and ethylcellulose and then foamed to obtain edible oleofoams (CP-EOs) as a solid-fat replacer. The average size of CP-PE droplets without surfactant was 598 ± 69 nm, as confirmed by confocal and transmission electron microscopy. Foaming with citric acid/NaHCO_3_ and structuring with ≥6% glyceryl monostearate resulted in CP-EOs with an overrun of 147 ± 4% and volumetric stability for 72 h. Micro-computed tomography showed a uniform microcellular network, while the rheological analysis showed solid-like behavior with a storage modulus higher than butter. Differential scanning calorimetry showed a melting enthalpy similar to unsalted butter (10.1 ± 0.9 J/g). These physicochemical properties demonstrate that CP-EOs can closely mimic the firmness, thermal profile, and mouth-feel of conventional solid fats and may provide a promising solid-fat replacer.

## 1. Introduction

Various types of solid fats, such as lard, beef tallow, butter, and hydrogenated vegetable oils, are widely used in baked goods, deep-fried foods, coffee creamers, and spreads. These fats, rich in saturated fatty acids or produced by hydrogenation, provide the characteristic texture and firmness that consumers expect [1]. However, consumption of these fats, especially partially hydrogenated oils containing trans-fats, has been shown to worsen blood lipid levels and increase the risk of cardiovascular disease [2]. Major international food and health organizations have taken a stricter stance on dietary saturated and trans fats. In 2015, the U.S. Food and Drug Administration (FDA) announced a final decision that partially hydrogenated oils, the main industrial source of trans fats, are not “generally recognized as safe (GRAS)” [3]. The World Health Organization (WHO) reinforced this stance by launching the “REPLACE” campaign, urging member states to enact laws and policies to eliminate industrially produced trans fats from the global food supply by 2023. Epidemiological studies have shown that replacing saturated fats with unsaturated oils reduces the incidence and mortality of coronary heart disease [4]. As a result, lipid saturation has become an important purchasing criterion for health-conscious consumers [2].

To meet these demands, oleofoams, air-in-oil dispersion systems, have attracted growing interest, even though many researchers have faced challenges in their production [5,6]. One obstacle is the inherently low surface tension of the immiscible liquid (~15–30 mN/m), which hinders bubble stabilization. Another one is the lack of food-grade stabilizers that are oil-dispersible and have sufficient surface activity at the oil–gas interface [6,7]. Nevertheless, direct addition of gas bubbles to edible oil can significantly reduce the energy density and total fat, saturated fat, and trans-fat content of fatty foods [7].

Unlike conventional emulsions, Pickering emulsions are stabilized by solid particles that are irreversibly adsorbed at the interface between immiscible fluids. This system exhibits excellent coalescence resistance and is less sensitive to environmental stresses such as pH, ionic strength, temperature, and oil composition due to its surfactant-free nature [8,9]. Cellulose, the most abundant natural polymer, is composed of β-1,4-linked glucose units and is generally recognized as safe. The native structure of cellulose contains amorphous and crystalline regions, and selective removal of the amorphous regions produces microcrystalline cellulose (MCC), which has already been commercialized as a thickening, gelling, and emulsion stabilizing agent in food and pharmaceutical industries [10,11]. Ethylcellulose (EC), a hydrophobic cellulose ether derived from cotton and wood pulp, is a free-flowing, biocompatible powder that is soluble in many organic solvents [12]. EC is approved as a food additive and is used as a stabilizer, thickener, and micro-encapsulation coating material. The Joint FAO/WHO Expert Committee on Food Additives has classified EC along with six related cellulose derivatives and has assigned an acceptable daily intake of “not specified”, considering its low toxicological concern [13]. Both MCC and EC are derived from abundant and renewable plant-based sources, making them sustainable and biodegradable alternatives to synthetic or animal-derived emulsifiers. The use of these cellulose derivatives is in line with global sustainability goals and regulatory guidelines of major food safety agencies such as the FDA and the European Food Safety Authority (EFSA). Therefore, cellulose-based Pickering emulsions are a promising strategy for food manufacturers seeking environmentally friendly and regulatory-compliant ingredients for healthier food formulations. Recent studies have shown that MCC, due to its rigid rod-like shape and high aspect ratio, effectively adsorbs to the oil–water interface, forming a dense interfacial layer that provides mechanical stability and steric hindrance to droplet coalescence. Meanwhile, EC has moderate hydrophobicity and film-forming ability, which can enhance particle affinity for oil and strengthen the interfacial network. MCC-based Pickering emulsions have been widely studied in the fields of pharmaceutical and food delivery [14], and EC has mainly been used as a coating or structuring agent in lipid systems [15]. However, there have been only a few studies on stabilizing air-in-oil foam by utilizing the synergistic effect of MCC and EC, especially in the field of edible oleofoam. This study aims to address these issues by developing a structurally stable and clean-labelable alternative to conventional solid fats by utilizing the complementary interfacial properties of MCC and EC.

Emulsions, which are dispersions of two immiscible liquids, are thermodynamically unstable and tend to separate over time through gravitational settling, flocculation, or coalescence. While conventional emulsifiers are amphiphilic molecules that lower the interfacial tension, the Pickering emulsion strategy used in this study was used to prepare oleofoams stabilized solely by edible cellulose particles [16]. Previous studies have used inorganic or synthetic particle stabilizers, but few studies have used edible cellulose derivatives for oleofoam formation.

Therefore, the objective of this study was to prepare edible oleofoams, air-in-oil dispersions stabilized with MCC and EC particles as a healthier alternative to saturated or hydrogenated solid fats, and to characterize their physicochemical properties. By demonstrating that cellulose Pickering particles can overcome the common barriers to oleofoam stability, a novel and practical approach toward solid-fat replacers was provided to meet both regulatory requirements and consumer demands for healthier foods.

## 2. Results and Discussion

### 2.1. Mean Particle Diameter and Polydispersity Index (PDI) of the Cellulose Particle-Based Pickering Emulsions (CP-PEs)

**Figure 1:**
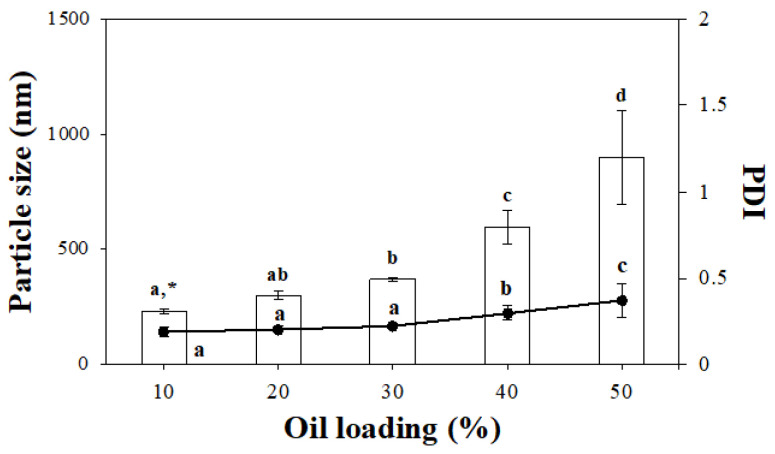
Effect of oil content on the mean particle diameter and PDI of the CP-PEs. * Different letters indicate a significant difference at *p* < 0.05 (*n* = 3).

Ultrasonication treatment is known to be one of the most effective methods for preparing stable emulsion systems [17]. Cellulose particle-based Pickering emulsions (CP-PEs) were prepared by ultrasonication. As shown in Figure 1, the mean particle diameter and polydispersity index (PDI) of the CP-PEs varied from 232.52 ± 11.75 nm to 899.70 ± 200.72 nm and from 0.19 ± 0.03 to 0.37 ± 0.09, respectively. The mean particle size and PDI of the CP-PEs increased with increasing oil content, and the optimum oil content was determined to be 40%. It is well established that the stability of Pickering emulsions depends on many parameters such as oil/water ratio, particle concentration, pH, and ionic strength [18]. Consistent with a previous report [19], the oil/water ratio significantly (*p* < 0.05) affected the particle size observed in this study. The mean particle diameter of CP-PEs with 40% oil content was 597.83 ± 68.8 nm, which was significantly smaller (*p* < 0.05) than that of CP-PEs with 50% oil content (899.70 ± 200.72 nm). This increase in particle size with increasing oil content could be due to the limited availability of cellulose particles to adequately stabilize the larger interface area generated by the higher oil fractions, which resulted in droplet coalescence and larger particle diameters. Further increase in oil content resulted in a drastic deterioration in emulsion instability, which was indicated by a significant (*p* < 0.05) increase in particle size and high PDI value (0.37 ± 0.09), reflecting a broad particle size distribution and agglomeration of particles. In contrast, CP-PEs with oil content up to 40% showed low PDI values (<0.3), indicating a very uniform and monodisperse size distribution [20]. Therefore, an oil content of 40% was considered optimal for producing stable cellulose particle-based edible oleofoams (CP-EOs). The optimal mean particle diameter (598 ± 69 nm) at 40% oil content was significantly (*p* < 0.05) smaller than those typically observed for soy lecithin-stabilized emulsions with droplet sizes in the range of 0.5–1.0 μm under similar conditions [21] and lower than those reported for Pickering systems stabilized with inorganic particles with particle sizes in the micrometer range [19]. This highlights the enhanced effectiveness of the cellulose-based particle stabilizers used in this study.

### 2.2. Morphological Characterization of the CP-PEs

#### 2.2.1. Optical Images of the CP-PEs

**Figure 2:**
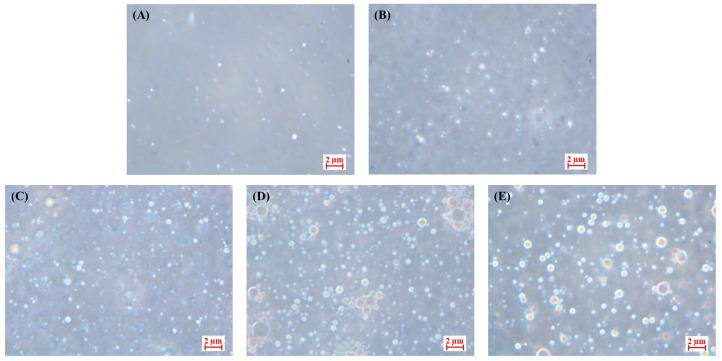
Optical microscopy images of CP-PEs with different oil contents. (**A**) 10%, (**B**) 20%, (**C**) 30%, (**D**) 40%, and (**E**) 50%.

Figure 2 shows the optical microscope images of CP-PEs with different oil contents (10–50%). The images generally show round and smooth particles. As the oil content increased from 10% to 40%, the particle density increased noticeably, and the particles appeared well-dispersed and relatively uniform, which is in good agreement with the mean diameter and the PDI results discussed above. However, when the oil content reached 50%, the particle density decreased noticeably, and the particle sizes became more heterogeneous. This indicates that the insufficient covering of cellulose-based particles at high oil fractions increases the instability and leads to significant particle aggregation or coalescence, resulting in rapid phase separation and formation of an oil layer.

#### 2.2.2. Structure of Oil and Cellulose Particles in the CP-PEs

**Figure 3:**
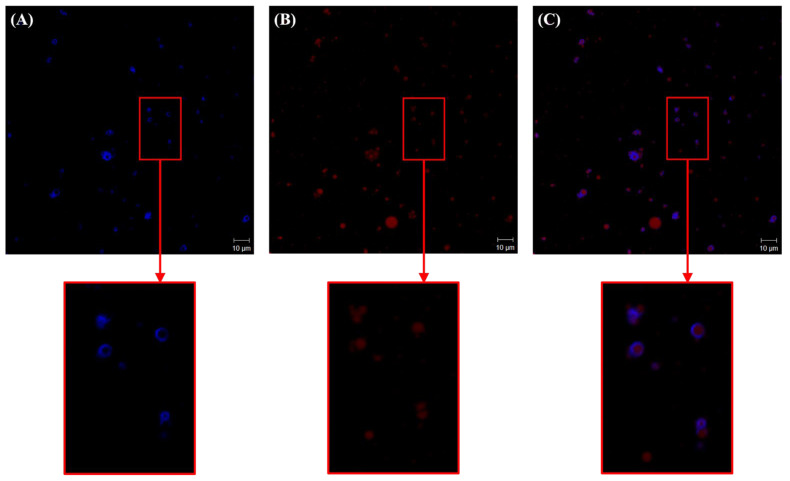
CLSM images of CP-PEs with an oil content of 40%. (**A**) cellulose channel, (**B**) oil phase channel, and (**C**) merged channel.

Confocal laser scanning microscopy (CLSM) was used to visualize the microstructure of CP-PEs and confirm the Pickering-type stabilization mechanism. CLSM has been previously effectively applied to characterize Pickering emulsions stabilized by various solid particles, including gelatin [22], starch [23], cellulose [24], and complex biopolymer particles [25]. In this study, the cellulose particles were labeled with blue fluorescence, while the oil droplets were labeled with red fluorescence. As shown in Figure 3, the blue fluorescence corresponding to the cellulose particles clearly surrounds the red fluorescent oil droplets, providing direct evidence of the Pickering emulsion structure. The merged image (Figure 3C) further confirms that the oil droplets are effectively stabilized by the cellulose particles adsorbed at the oil–water interface. Additionally, the presence of independently suspended cellulose particles (blue fluorescence in the continuous aqueous phase) suggests the formation of multilayers of cellulose particles on the droplet surface or the presence of excess cellulose particles that are not adsorbed at the interface, which may be due to the high particle concentration in the formulation [26]. These CLSM results support the morphological observations discussed previously, confirming that CP-PEs were successfully stabilized by the cellulose-based Pickering particles.

#### 2.2.3. Structure of Microcrystalline Cellulose (MCC) and Ethyl Cellulose (EC) in the CP-PEs

**Figure 4:**
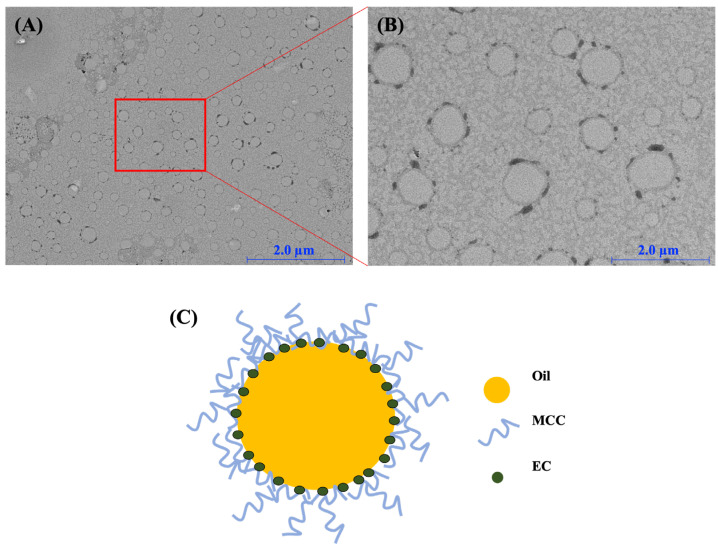
TEM images of CP-PEs with an oil content of 40% (**A**) at a magnification of 5.0 K and (**B**) at a magnification of 20.0 K. (**C**) Schematic illustration of the synergistic stabilization mechanism of CP-PEs by MCC and EC.

The morphology of CP-PEs prepared with 40% grapeseed oil was observed by transmission electron microscopy (TEM). The CP-PE particles appeared spherical and had smooth surfaces. The samples were negatively stained with a phosphotungstic acid hydrate solution, which allowed for the visualization of MCC and EC as relatively dark regions and distinct dark spots, respectively [27,28]. As shown in Figure 4, the dispersed oil phase was clearly surrounded by dark regions and spots, indicating that MCC and EC were effectively adsorbed at the oil–water interface. In addition, the dark regions representing MCC were evenly distributed in the continuous aqueous phase of CP-PEs, suggesting the presence of excessive MCC particles. Therefore, the TEM results clearly confirmed the Pickering emulsion structure of CP-PEs. These results suggest that MCC and EC play complementary roles in stabilizing the oil–water interface. MCC, with its high aspect ratio and stiffness, forms a dense and continuous network around the oil droplets, providing mechanical strength and inducing steric hindrance for coalescence. Meanwhile, EC, due to its hydrophobicity, can be adsorbed at the interface, enhancing the affinity between the cellulose-based particles and the oil phase, thereby reinforcing the interfacial film. The coexistence of the two particles appears to support the synergistic stabilization effect by forming an interfacial barrier with stronger cohesion than either component alone. The proposed interfacial arrangement is conceptually illustrated in Figure 4C to help understand the dual stabilization mechanism provided by MCC and EC.

### 2.3. Characterization of the Cellulose Particle-Based Edible Oleofoams (CP-EOs)

#### 2.3.1. Overrun of the CP-EOs with Different Concentrations of NaHCO_3_

CP-EOs were prepared by direct addition of aqueous citric acid solution to CP-PEs. Traditionally, edible oleofoams prepared from solid lipid materials have been mechanically aerated using the whipping method [29,30,31]. However, in this study, CP-EOs were aerated chemically by the reaction of citric acid and sodium bicarbonate (NaHCO_3_). Various concentrations of NaHCO_3_ were added to the CP-PE formulation prior to foaming, and the overrun was calculated by comparing the heights of the initial CP-PEs and the resulting CP-Eos, as described in Section 4.6.1.

**Figure 5:**
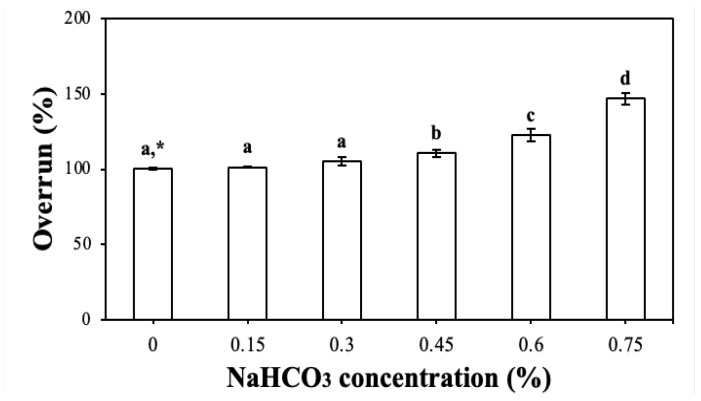
Overrun of the CP-EOs with different NaHCO_3_ concentrations. * Different letters indicate a significant difference at *p* < 0.05 (*n* = 3).

**Figure 6:**
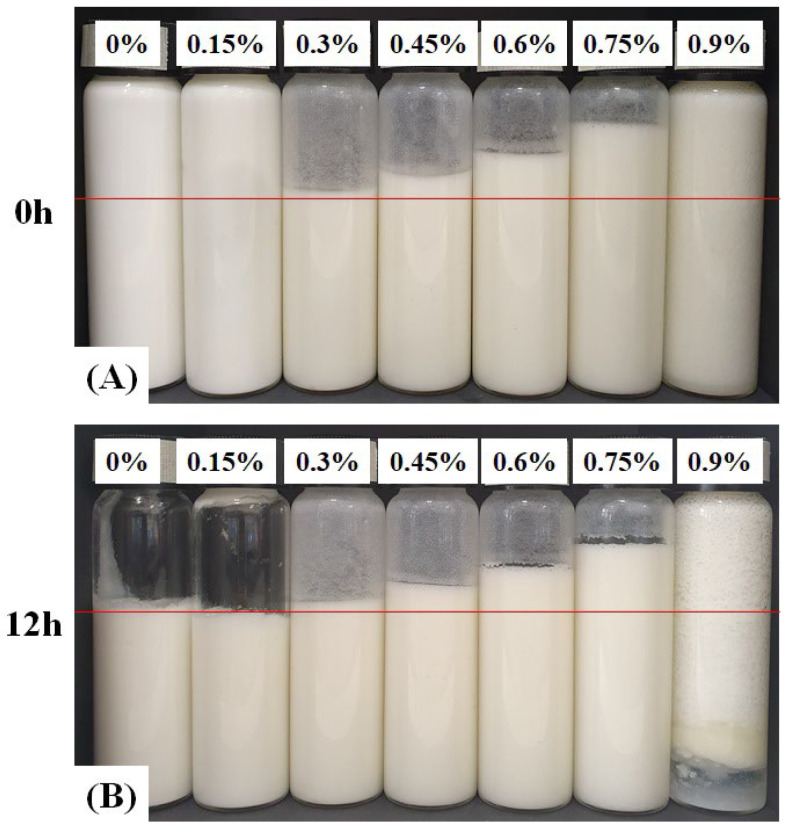
Appearance of the CP-EOs with different NaHCO_3_ concentrations. (**A**) At initial and (**B**) stored for 12 h.

As shown in Figure 5, the overrun of CP-EOs showed a clear positive correlation with the NaHCO_3_ concentrations, increasing from 100.27 ± 0.47% for 0% NaHCO_3_ to 146.85 ± 4.03% for 0.75% NaHCO_3_. The CP-EOs prepared with NaHCO_3_ concentrations ranging from 0% to 0.75% remained stable and uniform even after 12 h of storage, as shown in Figure 6. In particular, the 0% and 0.15% NaHCO_3_ groups showed very low wall adhesion and faster volume loss, which is likely due to the weak foam structure and incomplete droplet expansion caused by insufficient CO_2_ production. This explains why there is a noticeable visual difference compared to the other formulations. However, at a higher concentration (0.9% NaHCO_3_), a noticeable phase separation occurred after 12 h, suggesting that excessive NaHCO_3_ may have compromised the foam stability by increasing bubble coalescence or collapse [18]. Therefore, the optimal NaHCO_3_ concentration that did not compromise the stability of CP-EOs was determined to be 0.75% to provide maximum overrun. Overrun was linearly increased up to 0.75% NaHCO_3_ concentration due to the increased CO_2_ gas production caused by the reaction of citric acid and NaHCO_3_. According to the stoichiometry of this reaction, approximately 0.22 mL of CO_2_ is released per gram of NaHCO_3_ at standard conditions (25 °C and 1 atm) [32,33]. Therefore, bubble nucleation and foam expansion are limited by the amounts of reactants up to this concentration. Above 0.75%, excessive CO_2_ production exceeds the stabilizing capacity of the cellulose particles at the interface, promoting bubble coalescence and subsequent foam collapse, as evident by the significant phase separation at 0.9% (Figure 6B). This behavior is consistent with previous observations reported in chemically aerated oleogel systems [33].

#### 2.3.2. Overrun and Volumetric Stability of CP-EOs with Different Concentrations of Glyceryl Monostearate (GMS)

**Figure 7:**
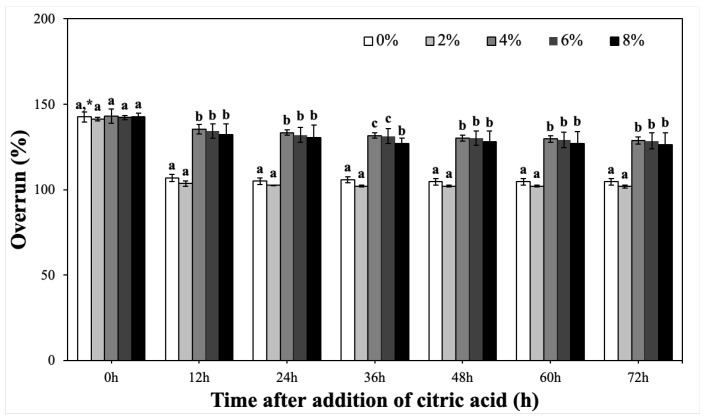
Overrun of CP-EOs with different GMS concentrations and times after adding citric acid. * Different letters in the same time indicate significant differences at *p* < 0.05 by Duncan’s multiple range test.

CP-EOs were prepared with various concentrations of glyceryl monostearate (GMS) and stored at ambient temperature for 72 h to investigate the effect of GMS on the volume stability of CP-EOs. As a result, the volume stability of CP-EOs was significantly improved as the GMS concentration increased. However, as shown in Figure 7, the initial overrun values (0 h) did not show significant differences among the various GMS concentrations. After 12 h of storage, the overrun values of CP-EOs containing low GMS concentrations (0%, 2%, and 4%) significantly (*p* < 0.05) decreased, whereas CP-EOs containing 6% and 8% GMS maintained relatively high and stable overrun values. In particular, CP-Eos, containing 6% GMS, showed the smallest volume change after 24 h and maintained stability during the storage period up to 72 h.

**Figure 8:**
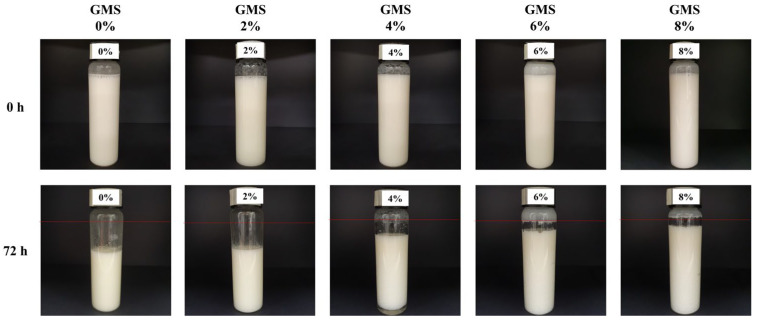
Appearance of the CP-EOs with different GMS concentrations after 0 h and 72 h.

As shown in Figure 8, CP-EOs prepared with 0% and 2% GMS showed rapid volume loss after 72 h of storage, suggesting poor long-term stability. Therefore, the optimal GMS concentration to obtain stable CP-EOs while minimizing oleogelator was determined to be 6%. These results are consistent with previous studies on oleogel-based emulsions, which reported that wax concentrations higher than 5% exhibited solid-like behavior and improved structural stability, making them suitable for use as shortening replacements or highly plasticized fats [34]. Similarly, the 6% GMS used in this study was shown to provide sufficient interfacial and volumetric stability, supporting its suitability as a fat replacement.

#### 2.3.3. Microstructure of the CP-EOs

**Figure 9:**
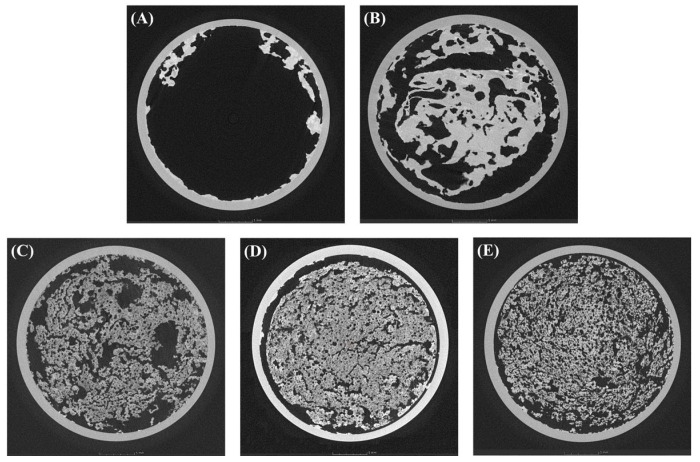
Micro-computed tomography (Micro-CT) images of the CP-EOs with different GMS concentrations. (**A**) 0%, (**B**) 2%, (**C**) 4%, (**D**) 6%, and (**E**) 8%.

The formation of the micro-cellular structure of CP-EOs was triggered by the reaction of citric acid and NaHCO_3_. However, the micro-cellular structure of CP-EOs was unstable in the absence of the oleogelator, because the carbon dioxide gas bubbles easily escaped and the structure collapsed quickly. In order to stabilize the micro-cellular structure of CP-EOs, GMS was added as an oleogelator during the preparation. As shown in Figure 9, CP-EOs prepared with low concentrations of GMS (0%, 2%, and 4%) could not effectively maintain a firm and uniform micro-cellular structure. In contrast, CP-EOs containing more than 6% GMS effectively trapped carbon dioxide gas bubbles within the structure to form a stable and uniform micro-cellular network. These observations are supported by previous studies that have highlighted the important role of crystal structure in stabilizing the oleofoam morphology. Du et al. [29] demonstrated that increasing the GMS concentration enhanced partial coalescence and strengthened the crystal network, which were essential for maintaining foam stability during whipping. Similarly, Metilli et al. [35] reported that controlling the crystallization conditions of cocoa butter-based oleofoams significantly affected the bubble size distribution and mechanical robustness of the foam. Their results confirmed that a dense and well-distributed crystal network could improve foam integrity by physically supporting the gas interface and suppressing bubble collapse. These results clearly indicate that a minimum GMS concentration of approximately 6% is required to obtain a stable CP-EO microstructure. It is supported by previous studies using solid lipids and mechanical whipping methods for oleofoam formation.

#### 2.3.4. Rheological Properties of the CP-EOs

**Figure 10:**
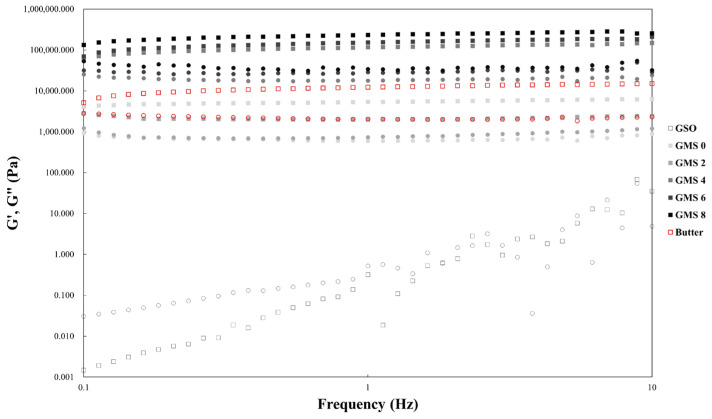
Frequency sweeps of unsalted butter and the CP-EOs with different GMS concentrations. The square and circle represent G′ and G″, respectively.

The rheological properties of the lyophilized CP-EOs were evaluated using frequency sweep. Figure 10 shows the frequency-dependent behavior of CP-EOs prepared with different GMS concentrations compared to unsalted butter as a reference. As shown in Figure 10, all samples showed a clear frequency dependence, with the storage moduli (G′) slightly increasing and the loss moduli (G″) slightly decreasing with increasing applied frequency. This behavior is characteristic of a viscoelastic solid. A previous system also showed similar trends except the values (around 100 Pa) of G′ of pea protein and different polysaccharides-based high internal phase emulsions (HIPE), which were much lower than those of CP-EOs [36]. However, other oleogels prepared by mixing whey protein concentrate (WPC) and basil seed gum (BSG) or xanthan gum (XG) showed a similar G′ value of 100,000 Pa to that of CP-EOs [37]. Moreover, the G′ significantly (*p* < 0.05) increased with increasing GMS concentration, indicating increased structural strength and reduced elastic deformation under applied stress. CP-EOs containing 4% or more GMS had higher G′ values than unsalted butter, indicating higher structural rigidity of these formulations. All CP-EO samples had G′ values higher than G″ values, indicating that solid-like properties (elastic behavior) dominate the rheological characteristics. A previous study on whipped cream has shown that the frequency-dependent behavior and dominant elastic properties of oleofoam suggest a highly viscoelastic structure [38].

#### 2.3.5. Melting Parameters of the CP-EOs

Table 1 shows the melting parameters obtained from differential scanning calorimetry (DSC) analysis. The melting parameters of CP-EOs prepared without GMS were not detectable. As shown in Table 1, the melting temperature (*T_m_*) increased significantly (*p* < 0.05) with increasing GMS concentration. However, the onset melting temperature (onset *T_m_*) did not show a significant difference (*p* > 0.05) among CP-EOs with respect to GMS concentration, which is likely because GMS is a single-component substance showing consistent initial melting behavior. The melting enthalpy (Δ*H_m_*) values increased significantly (*p* < 0.05) with increasing GMS concentration. The Δ*H_m_* value of unsalted butter (10.10 ± 0.87 J/g) was located between those of CP-EOs prepared with 6% GMS (8.44 ± 0.26 J/g) and 8% GMS (12.71 ± 0.54 J/g). The obtained enthalpy range of 8.44–12.71 J/g was almost identical to that of unsalted butter and also similar to that of palm-based shortenings manufactured from high-oleic palm oil reported by Perez-Santana et al. [39]. This suggests that CP-EOs can reduce saturated fat content by approximately 33% compared to conventional shortening while providing similar texture and melting characteristics suitable for bakery applications.

## 3. Conclusions

In this study, CP-PEs were successfully prepared using cellulose materials (MCC and EC) as Pickering particles, and the particle diameter was uniform with a mean diameter of 597.83 ± 68.83 nm. The structural characteristics of CP-PEs as Pickering emulsions were confirmed by CLSM and TEM, which clearly showed that MCC and EC particles densely surrounded oil droplets. CP-EOs were successfully prepared using CP-PEs as a template and NaHCO_3_ as a chemical blowing agent. The optimal CP-EO formulation showed an overrun of 146.85 ± 4.03% and maintained volume stability for 72 h at ambient temperature. Micro-CT imaging confirmed that CP-EOs with GMS concentration higher than 6% had a stable micro-cellular structure. Rheological analysis results showed that CP-EOs mainly exhibited solid-like behavior, and formulations with GMS concentration higher than 4% exhibited higher storage moduli (G′) values than unsalted butter. In addition, DSC analysis results showed that the Δ*H_m_* of CP-EOs with 6% and 8% GMNS were 8.44 ± 0.26 J/g and 12.71 ± 0.54 J/g, respectively, which were very close values of unsalted butter (10.10 ± 0.87 J/g). Consequently, these physicochemical properties strongly suggest that the developed CP-EOs can effectively mimic and replace conventional solid fats in various food applications such as bakery, spreads, and ice cream, and provide potential nutritional benefits by lowering saturated fat content. Although the rheological and thermal results suggest a butter-like mouthfeel, the lack of sensory and texture analysis and validation in real or model food systems remains a limitation. Future studies should include sensory evaluation, instrumental texture testing, and application-specific evaluations to validate functionality. While this study focused on short-term characterization, further studies are needed to evaluate long-term storage stability and heat resistance, which are essential for commercial implementation.

## 4. Materials and Methods

### 4.1. Materials

Sodium hydroxide (NaOH, 98.0%) was purchased from Samchun Chemicals (Pyeongtaek, Republic of Korea). MCC, EC (48% ethoxyl), Nile red, and calcofluor white stain were purchased from Sigma-Aldrich (St. Louis, MO, USA). Grapeseed oil (GSO, Ottogi Corp, Anyang, Republic of Korea) and unsalted butter (Anchor, Auckland, New Zealand) were purchased from a local market in Seoul, Republic of Korea. GMS was obtained from Daejung Chemicals (Seoul, Republic of Korea). Prethanol A (PetOH, 96%) was purchased from Duksan reagents (Ansan, Republic of Korea). NaHCO_3_ (sodium bicarbonate, <99%) and citric acid monohydrate (100%) were purchased from Serimfood (Bucheon, Republic of Korea). Phosphotungstic acid hydrate was purchased from Fisher Scientific Korea Ltd. (Incheon, Republic of Korea). All other chemicals used were of analytical grade.

### 4.2. Preparation of CP-PEs

CP-PEs were prepared using a probe-type of ultrasonication (VCX-750, Sonics & Materials Inc., Sandy Hook, CT, USA), and the formulations are summarized in Table 2. To prepare the aqueous phase, accurately weighed MCC was suspended in distilled water (DW) and stirred at 50 °C, at 300 rpm for 40 min. EC was dissolved in PetOH at 500 rpm for 1 h. The EC solution was then poured into an equal mass of MCC suspension with vigorous stirring. The aqueous phase and GSO were placed in a glass vial and kept in a water bath at 50 °C to prevent gelation of the cellulose. Ultrasonication was performed in an ice bath to avoid overheating the sample. The power level was varied by 3 s of sonication with 2 s of standby for 20 min. The CP-PEs were then characterized as quickly as they were prepared.

### 4.3. Mean Droplet Size and PDI of the CP-PEs

The mean particle size and PDI of the CP-PEs were measured by dynamic light scattering (DLS) using a zeta potential and mean particle diameter analyzer (ELSZ-2000, Otsuka Electronics Co., Ltd., Osaka, Japan) at room temperature (RT). Prior to measurement, the sample was diluted 100-fold with distilled water to reduce turbidity, minimize multiple scattering, and prevent detector saturation to ensure accurate DLS measurements. This was confirmed through preliminary optimization tests.

### 4.4. Morphological Characterization of the CP-PEs

#### 4.4.1. Optical Microscopy

Optical micrographs of the CP-PEs were captured by an optical microscope (BX-43, Olympus Corp., Tokyo, Japan) equipped with a digital camera (IMTcamCCD PRO2, IMT Inc., North Hollywood, CA, USA). The sample was placed directly on a microscope slide and observed at 100× magnification.

#### 4.4.2. Confocal Light Scanning Microscopy (CLSM)

CLSM was performed to visualize oil droplets and cellulose materials of CP-PEs. CLSM images of the samples were captured using a confocal microscope (LSM700, Carl Zeiss Microimaging GmbH, Jena, Germany). Exactly 500 μL of the sample was transferred to a 1.5 mL tube and mixed with 10 μL of Nile Red (0.1% *w*/*v* in dimethyl sulfoxide, excitation 514 nm, emission 539–648 nm) and 100 μL of calcofluor white (1.0% *w*/*v* in DW, excitation 405 nm, emission 410–523 nm). The mixture was vortexed for 10 s and equilibrated for 10 min, and then 30 μL was placed on a concave slide. The sample was covered with a coverslip and observed using an oil immersion objective lens at 63× magnification.

#### 4.4.3. Transmission Electron Microscopy (TEM)

The morphology of CP-PEs was analyzed using TEM (HT 7700, Hitachi, Tokyo, Japan). Briefly, 1% (*v*/*v*) CP-PEs were diluted in DW, and 20 μL of the diluted solution was placed on a carbon-coated copper grid (Cu, 200 mesh, Ted Pella, Redding, CA, USA). One drop of 1% (*w*/*v*) phosphotungstic acid hydrate (Alfa Aesar, Haverhill, MA, USA) was added as a negative stain. After incubation at ambient temperature for 30 s, the excess liquid was removed using a piece of qualitative filter paper. The grid was air-dried, and images were recorded at 5.0 K and 20.0 K magnifications at a voltage of 100 kV.

### 4.5. Preparation of CP-EOs

The preparation method for CP-EOs was similar to that of CP-PEs described in Section 4.2, with modifications detailed as follows: NaHCO_3_ was additionally included in the aqueous phase. To prepare the oil phase, accurately weighed GMS was added as an oleogelator to a gently stirring GSO, heated to 90 °C. The CP-PEs obtained were equilibrated at 30 °C for 30 min, after which 1 mL of 18% (*v*/*w*) citric acid solution was added, followed by vigorous hand-shaking to induce foaming. Finally, the samples were frozen at −80 °C for 1 day and lyophilized to remove water and ethanol. The detailed formulations for CP-EOs are presented in Table 3 and Table 4.

### 4.6. Characterization of the CP-EOs

#### 4.6.1. Overrun of the CP-EOs

The overrun of the CP-EOs was investigated according to the method described in a previous study [29], with slight modifications. CO_2_ gas bubbles were incorporated into the CP-PEs by the reaction of NaHCO_3_ and citric acid, which increased the height of the sample in the vial. The overrun (%) of CP-EOs was calculated using the following Equation (1):

(1) $$Overrun\;\left(\%\right)=\frac{h_{c}}{h_{i}}\times 100$$

where h_i_ is the initial height of the CP-PEs and h_c_ is the height of the foamed CP-PEs after the addition of citric acid solution. The h_c_ was measured when the height of the sample did not increase after the addition of citric acid solution. The overrun of CP-EOs with various GMS concentrations was examined from 0 h to 72 h.

#### 4.6.2. Micro-Computed Tomography (Micro-CT) Analysis

Micro-CT was used to obtain X-ray images of the cross-sections of lyophilized CP-EOs. The samples were scanned using a high-resolution desktop X-ray Micro-CT system (XT H 225, Nikon Corp., Tokyo, Japan) operating at 100 kV voltage and 96 μA current. The X-ray detector consisted of a 12-bit digital cooled CCD camera with 1024 × 1024 pixels. The magnification was determined as the ratio of the distance from the tube to the detector to the distance to the object and ranged from 10× to 120×.

#### 4.6.3. Rheological Property of the CP-EOs

The rheological measurements of CP-EOs were performed using a rheometer system (HAAKE Rheostress 1, Thermo Fisher Scientific Inc., Waltham, MA, USA). A parallel plate cross-hatched geometry with a diameter of 35 mm was used, and the geometric gap was set at 1000 μm. Frequency sweeps (0.1–100 Hz, stress = 10 Pa) of unsalted butter and CP-EOs were used at 25 °C.

#### 4.6.4. Differential Scanning Calorimetry (DSC) Analysis

Thermal properties were analyzed using DSC (DSC25, TA Instruments Co., New Castle, DE, USA). A total of 5–10 mg of CP-EOs was weighed and placed in a DSC pan and hermetically sealed. The prepared sample was placed in the DSC cell, and nitrogen gas was purged at a flow rate of 50 mL/min to maintain an inert atmosphere. The sample was cooled down to −40 °C at 5 °C/min and equilibrated for 2 min. Thermograms were recorded by heating up to 90 °C at 5 °C/min, equilibrated for 2 min, and then cooled down to 0 °C at 5 °C/min. Indum and an empty DSC pan were used as calibration and reference, respectively.

### 4.7. SPSS Analysis

Experiments were performed in triplicate, and data were expressed as the mean ± standard deviation (SD). Statistical analysis was performed using SPSS version 26.0 (SPSS Inc., Chicago, IL, USA). Significant differences among treatments were determined by analysis of variance (ANOVA) and Duncan’s multiple range test. The level of significance was set at *p* < 0.05.

## Figures and Tables

**Table 1:** Thermal parameters of unsalted butter and CP-EOs with different concentrations of GMS.

Sample (GMS Concentrations)	*T_m_* (°C)	Onset *T_m_* (°C)	Δ*H_m_* (J/g)
Unsalted butter	14.07 ± 0.06 ^a,^*	10.18 ± 0.01 ^a^	10.10 ± 0.87 ^d^
CP-EOs (0%)	ND **	ND	ND
CP-EOs (2%)	43.40 ± 0.32 ^b^	39.96 ± 0.72 ^b^	1.32 ± 0.27 ^a^
CP-EOs (4%)	47.37 ± 0.01 ^c^	40.54 ± 0.12 ^b^	5.43 ± 0.58 ^b^
CP-EOs (6%)	48.02 ± 0.11 ^d^	40.02 ± 0.20 ^b^	8.44 ± 0.26 ^c^
CP-EOs (8%)	48.90 ± 0.11 ^e^	40.09 ± 0.20 ^b^	12.71 ± 0.54 ^e^

* Different letters in the same column indicate a significant difference at *p* < 0.05 (*n* = 3) by Duncan’s multiple range test. ** Not detectable.

**Table 2:** Ratio of oil, cellulose materials, distilled water, and PetOH for preparing the CP-PEs.

Sample (Oil Concentrations)	Oil Phase	Aqueous Phase			
	GSO (g)	MCC (g)	EC (g)	DW (g)	PetOH (g)
CP-PEs (10%)	3	0.54	0.54	12.96	12.96
CP-PEs (20%)	6	0.48	0.48	11.52	11.52
CP-PEs (30%)	9	0.42	0.42	10.08	10.08
CP-PEs (40%)	12	0.36	0.36	8.64	8.64
CP-PEs (50%)	15	0.30	0.30	7.20	7.20

**Table 3:** Ratio of GSO, GMS, cellulose materials, DW, PetOH, and NaCO_3_ for preparing the CP-EOs with different NaHCO_3_ concentrations.

Sample (NaCO_3_ Concentrations)	Oil Phase		Aqueous Phase				
	GSO (g)	GMS (g)	MCC (g)	EC (g)	DW (g)	PetOH (g)	NaHCO_3_ (g)
CP-EOs (0.00%)	10.2	1.8	0.36	0.36	8.640	8.640	0.000
CP-EOs (0.15%)					8.595		0.045
CP-EOs (0.30%)					8.550		0.090
CP-EOs (0.45%)					8.505		0.135
CP-EOs (0.60%)					8.460		0.180
CP-EOs (0.75%)					8.415		0.225

**Table 4:** Ratio of GSO, GMS, cellulose materials, DW, PetOH, and NaCO_3_ for preparing the CP-EOs with different GMS concentrations.

Sample (GMS Concentrations)	Oil Phase		Aqueous Phase				
	GSO (g)	GMS (g)	MCC (g)	EC (g)	DW (g)	PetOH (g)	NaHCO_3_ (g)
CP-EOs (0%)	12.0	0.0	0.36	0.36	8.415	8.640	0.225
CP-EOs (2%)	11.4	0.6					
CP-EOs (4%)	10.8	1.2					
CP-EOs (6%)	10.2	1.8					
CP-EOs (8%)	9.6	2.4					

## Data Availability

The data presented in this study are available on request from the corresponding author. The data are not publicly available due to ethical.
